# Molecular mechanisms of dysfunction of muscle fibres associated with Glu139 deletion in *TPM2* gene

**DOI:** 10.1038/s41598-017-17076-9

**Published:** 2017-12-01

**Authors:** Yurii S. Borovikov, Nikita A. Rysev, Olga E. Karpicheva, Vladimir V. Sirenko, Stanislava V. Avrova, Adam Piers, Charles S. Redwood

**Affiliations:** 1 Institute of Cytology, Russian Academy of Sciences, 4 Tikhoretsky Avenue, St. Petersburg, 194064 Russia; 2 Radcliffe Department of Medicine, University of Oxford, John Radcliffe Hospital, Oxford, OX3 9DU United Kingdom

## Abstract

Deletion of Glu139 in β-tropomyosin caused by a point mutation in TPM2 gene is associated with cap myopathy characterized by high myofilament Ca^2+^-sensitivity and muscle weakness. To reveal the mechanism of these disorders at molecular level, mobility and spatial rearrangements of actin, tropomyosin and the myosin heads at different stages of actomyosin cycle in reconstituted single ghost fibres were investigated by polarized fluorescence microscopy. The mutation did not alter tropomyosin’s affinity for actin but increased strongly the flexibility of tropomyosin and kept its strands near the inner domain of actin. The ability of troponin to switch actin monomers “on” and “off” at high and low Ca^2+^, respectively, was increased, and the movement of tropomyosin towards the blocked position at low Ca^2+^ was inhibited, presumably causing higher Ca^2+^-sensitivity. The mutation decreased also the amount of the myosin heads which bound strongly to actin at high Ca^2+^ and increased the number of these heads at relaxation; this may contribute to contractures and muscle weakness.

## Introduction

The muscle thin filament is a cooperative-allosteric system composed of a backbone of actin monomers with the troponin-tropomyosin (TN-Tpm) complex located lengthwise along the F-actin^1^. Cooperative interactions between Tpm, TN and actin regulate the contraction of muscle fibres in response to changes in [Ca^2+^]^2^. At low levels of Ca^2+^, TN interacts with actin to change the conformation of actin monomers and to switch them “off”^3,4^, and constrains Tpm in a position close to the outer domain of actin (“blocked position”)^5^. This is the so-called “OFF” state of the thin filament in which Tpm and actin^3,5^ inhibit the strong binding of myosin cross-bridges to actin and, consequently, the actin-activated myosin ATPase and muscle contraction^1^. When Ca^2+^ binds to TN, some actin monomers change their conformation to the “switched on”^3^ and Tpm moves towards the inner domain of actin^3,5^ exposing some of the myosin-binding sites, though part of the sites still remains covered (“closed position”)^1,5^. When the myosin heads strongly attach to the actin filament, Tpm takes a position over the inner domain of actin (“open position”)^5^ and the majority of actin monomers are “switched on”^3^, the thin filament transits to the so-called “ON” state. In this state Tpm fully exposes the myosin binding sites on F-actin^1,5^ and, consequently, activates the actin-activated myosin ATPase and initiates muscle contraction. Tpm thus plays a pivotal role in regulation of actin-myosin interaction^1,2^.

In skeletal muscles, there are three main Tpm isoforms, α, β and γ, which are encoded by the TPM1, TPM2 and TPM3 genes, respectively^2^. Point mutations in these genes give rise to a wide spectrum of muscle diseases^6^. Functional analyses have suggested that certain mutations cause a hypocontractile phenotype in which there is lower myofilament Ca^2+^-sensitivity, reduced sliding speeds in motility assay and reduced cross-bridge cycling rate. Other TPM mutations produce a higher Ca^2+^ sensitivity and a slightly higher maximum sliding speed in motility assay to give a hypercontractile phenotype^6,7^. An example of a hypercontractile mutation is the Glu139 deletion in β-Tpm (ΔE139Tpm) due to a point mutation in TPM2 gene. The molecular mechanisms underlying a high Ca^2+^-sensitivity caused by this mutation remain obscure.

The main goal of the present study was to investigate the effect of the E139 deletion on Ca^2+^-dependent changes in position of the Tpm during the ATPase cycle and the response of the myosin heads and actin to Tpm movement. We showed that the ΔE139 mutation increases flexibility of Tpm strands and “freezes” Tpm in a position close to the inner domains of actin throughout the ATPase cycle, and postulate that this may be one of the reasons for the higher Ca^2+^- sensitivity and induction of contractures and muscle fibre weakness.

## Results and Discussion

### The ΔE139 mutation in TPM2 increases the Ca^2+^-sensitivity of the actin-activated ATPase activity of myosin sufragment-1

**Figure 1:**
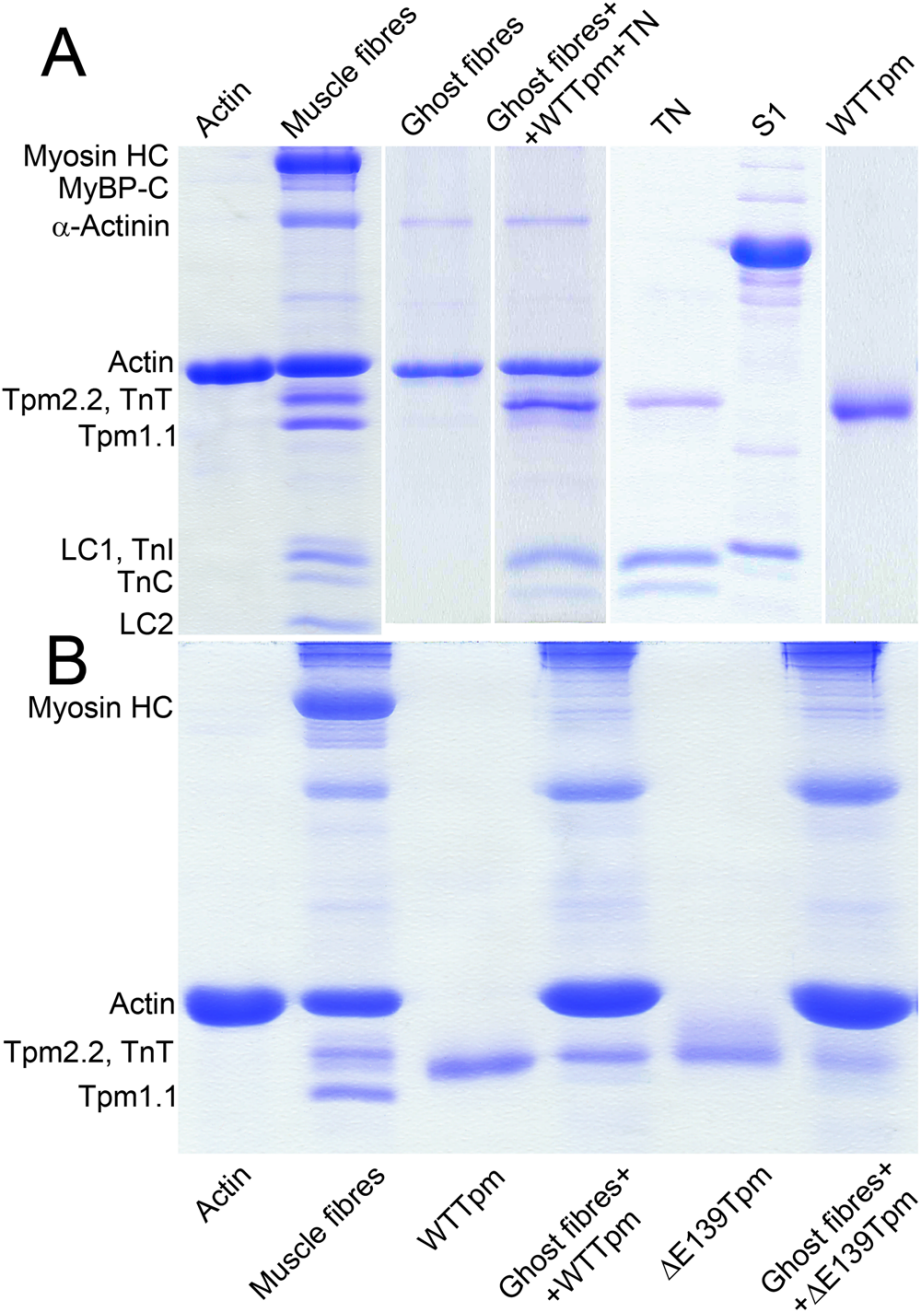
SDS-PAGE of muscle fibres (rabbit psoas) and proteins used in experiments. (**A**) Verification of myosin and Tpm-TN extraction from muscle fibres during the preparation of ghost fibres and reconstruction of the regulatory system with fast skeletal Tpm and TN. The image was assembled from several original gels (see Supplementary Figures A,B,C), aligned and cropped with the use of Adobe Photoshop CS5.1 software without any change in brightness and contrast. Ghost fibres do not contain Tpm1.1 and Tpm2.2, TN and myosin and consist of F-actin by more than 90%. Designations: Myosin HC – myosin heavy chains; LC1 and LC2 – myosin light chains; MyBP-C – myosin-binding protein C; TnT, TnI and TnC – troponin subunits. (**B**) Representative gel used for quantitative assessment of the ΔE139Tmp binding to actin filaments in ghost fibres as compared with the WTTpm (the full-length gel is presented as Supplementary Figure D). The ratio of WTTpm to ΔE139Tmp bound to actin estimated by ImageJ 1.48 software was 1: 0.85 ( ± 0.07). 8−10 fibres were used to prepare the probe loaded per gel. The unbound Tpm and TN were washed out by exposing the fibres to the washing solution for 15 min.

**Figure 2:**
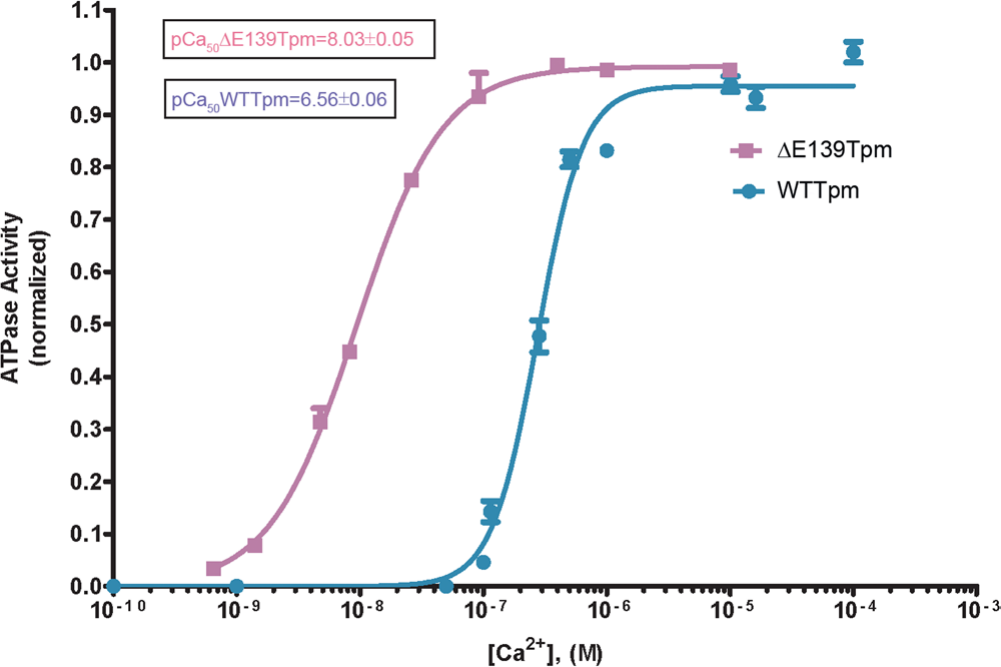
Effect of the E139 deletion in Tpm on sensitivity of the thin filaments to activating Ca^2+^ concentrations. Ca^2+^-dependence has been determined for fully regulated reconstituted thin filaments. The acto-S1 ATPase was measured in the presence of WTTpm (circles), and ΔE139Tpm (squares) at 25 °C. Error bars indicate ± SEM. pCa values were calculated from data averaged from 3 experiments. Conditions are given in Methods.

We first evaluated the effect of the Glu139 deletion in Tpm on Ca^2+^-sensitivity of the thin filaments in solution. The filaments were assembled with the wild-type tropomyosin (WTTpm) or ΔE139Tpm (Fig. 1) and used in measurements of actin-activated myosin S1 ATPase activity at increasing Ca^2+^ concentrations (see Methods). As indicated by a leftward shift of the pCa-ATPase curve obtained in the presence of the ΔE139Tpm, the mutation increased sensitivity of the thin filaments to Ca^2+^ (Fig. 2). The log of Ca^2+^ concentration required for half maximal activation of the ATPase activity (pCa_50_) were 8.03 ± 0.05 for filaments containing the ΔE139Tpm and 6.56 ± 0.06 for filaments reconstructed with the WTTpm (P < 0.01). Similar abnormally high Ca^2+^-sensitivity of the thin filaments containing ΔE139Tpm was previously detected by an *in vitro* motility assay^7^.

In our work with the ghost fibres, containing the WTTpm or ΔE139Tpm we mimicked different intermediate stages of the ATPase cycle at low (10^−9^ M) and high (10^−4^ M) Ca^2+^ concentrations.

### Ghost muscle fibres reconstituted with labelled proteins as a model for investigating conformational changes in proteins during the ATPase cycle

We have used a model system of thin filaments reconstituted in ghost muscle fibres using exogenous tropomyosin and troponin, decorated them with myosin subragment-1 (S1) and mimicked several steps of ATP hydrolysis cycle^3,4^. Prior to reconstitution, the ghost muscle fibres were completely devoid of tropomyosin, troponin and myosin (see Methods) (Fig. 1A). Exogenous tropomyosin (WTTpm or ΔE139Tpm), troponin and S1 were incorporated into the ghost fibres. Control experiments including SDS–PAGE analysis (Fig. 1B) and measurements of fluorescence intensity of the probes showed that the relative amount was practically the same for each of the incorporated proteins in all experiments.

In order to study the effect of the E139 deletion on the behavior of Tpm and the response of the myosin heads and actin to the movement of Tpm during the ATPase cycle we used polarized fluorimetry^8–11^; the polarized fluorescence of the studied protein reflects the average structural state of the population of protein molecules^3,4^. The AM state of the actomyosin complex was simulated in the absence of nucleotides. Mg-adenosine diphoshate (MgADP), Mg-adenosine 5′-(β,γ-imido)triphosphate tetralithium salt hydrate (MgAMP-PNP), and Mg-adenosine triphosphate (MgATP) were used to mimic the AM^·ADP, AM*·ADP, and AM**·ADP·Pi states, respectively^3,4^.

Tpm, S1 and F-actin were labelled with fluorescent probes: 5-iodoacetamidofluorescein (5-IAF) was covalently linked to cysteines of Tpm, N-(iodoacetaminoethyl)-1-naphthyl-amine-5-sulfonic acid (1,5-IAEDANS) was specifically linked to Cys707 of S1, and phalloidin-fluorescein isothiocyanate (FITC-phalloidin) was bound to F-actin in the region of actin groove (see Methods) which initiated polarized fluorescence (Supplementary Tables S1–S3) and allowed determining the changes in flexibility and spatial arrangement of the Tpm strands, myosin heads, and actin subunits in the muscle fibres, respectively.

**Figure 3:**
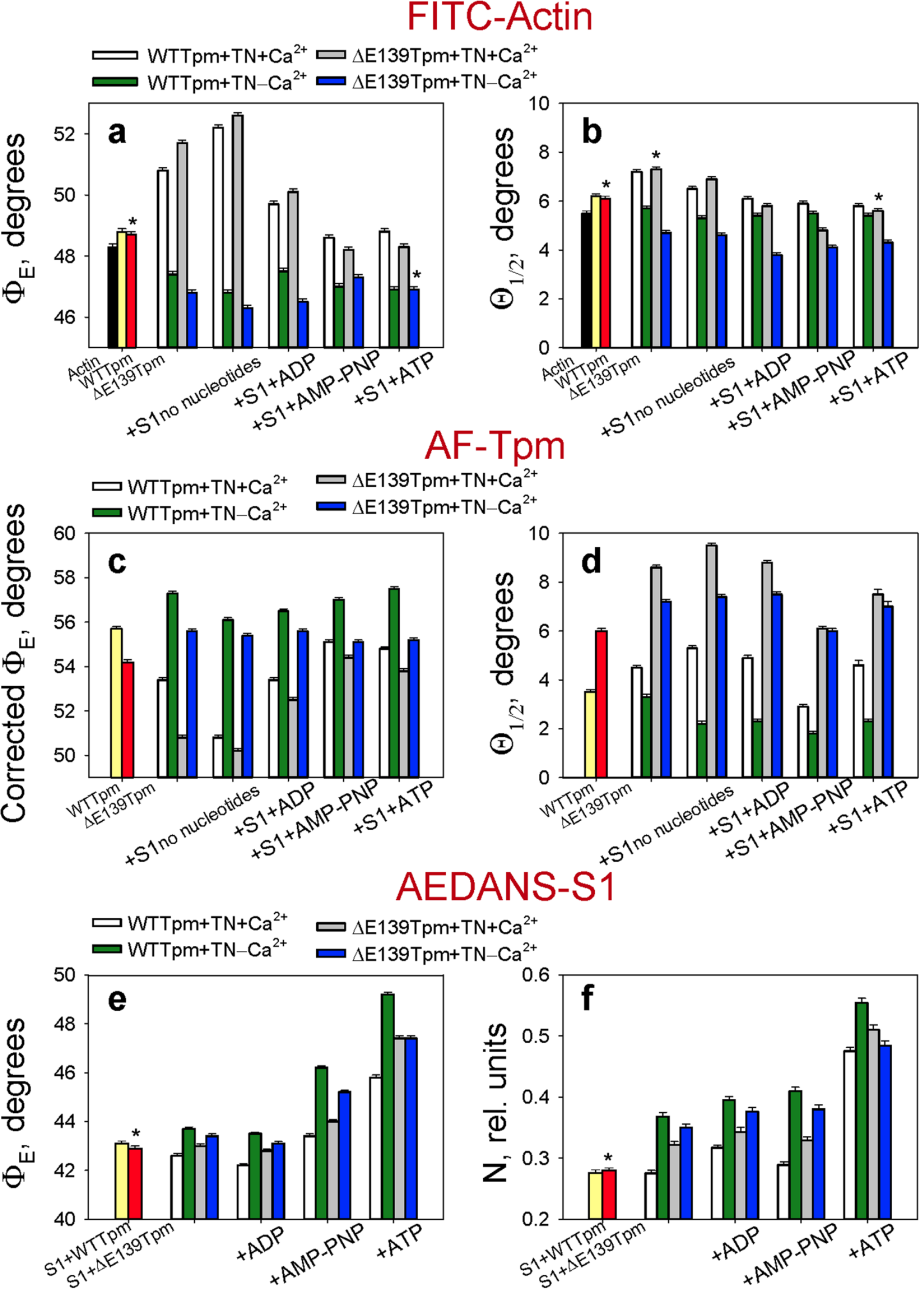
The effect of the E139 deletion in Tpm, Ca^2+^ and TN on the values of Ф_Е_ (**a,c,e**) and θ_1/2_ (**b,d,f**) of the polarized fluorescence of FITC-phallodin-actin (**a,b**), 5-IAF-labelled Tpm (**c,d**) and 1,5-IAEDANS-labelled S1 (**e,f**), respectively, at mimicking the different stages of the actomyosin ATPase cycle. The Ф_Е_ and θ_1/2_ values for the WTTpm and ΔE139Tpm are significantly altered by TN and Ca^2+^ (*p* < 0.05). Asterisks (*) indicate unreliable differences in the values between the WTTpm and ΔE139Tpm. Error bars indicate ± SEM.

In agreement with our earlier findings^3,4^, the values of the angle between the fibre axis and the emission dipole of the probe (Φ_E_) in the presence of WTTpm were found to be 48.8°, 55.7°, and 43.1° for FITC-actin, AF-WTTpm and AEDANS-S1, respectively. The exchange of WTTpm for ΔE139Tpm in its complex with F-actin, binding of S1and/or troponin to the F-actin-Tpm complex at high and low Ca^2+^ in the ghost fibres induced changes in the Φ_E_, θ_1/2_ and N values for all the labelled proteins (Fig. 3) that indicated conformational changes in F-actin, tropomyosin and the myosin heads^3,4^.

### The mutation preserves the troponin’s ability to switch actin monomers “off” and “on” at low and high Ca^2+^

**Figure 4:**
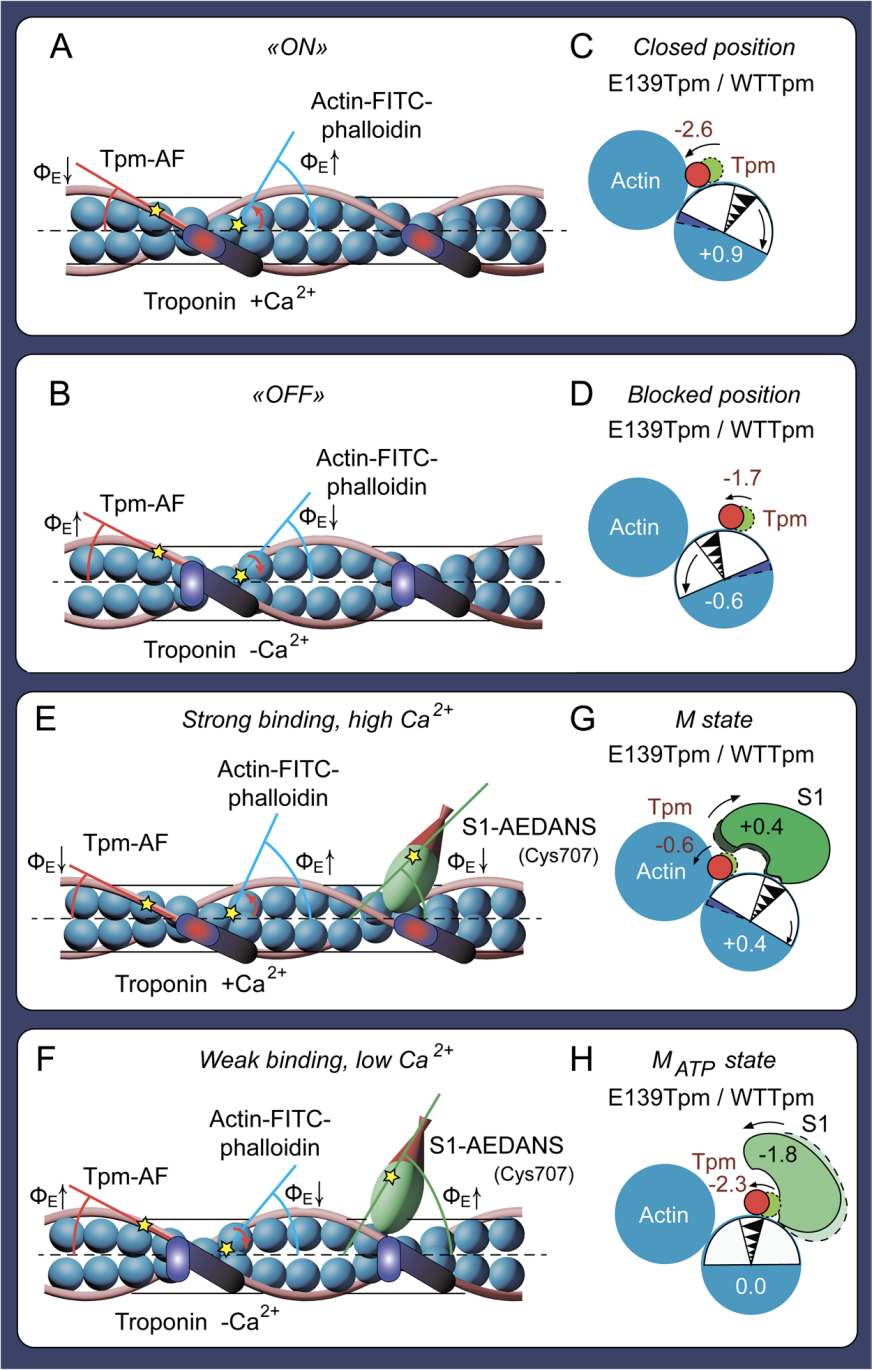
The presumed relationship between changes in the polarized fluorescence parameter Φ_E_ and spatial rearrangements of the proteins in the complex F-actin–Tpm–TN at high (**A**,**C**) and low (**B**,**D**) Ca^2+^, and in the complex F-actin–Tpm–TN–S1 at simulation of strong (**E**,**G**) and weak (**F**,**H**) binding of S1 to F-actin. Fluorescent probes are denoted as yellow stars. Changes in the Φ_E_ values (in degrees) for F-actin-FITC-phalloidin, Tpm-AF and S1-AEDANS induced by E139 deletion are designated (**A,B,E,F**) and corresponding spatial rearrangements of actin monomers, Tpm and the myosin heads are depicted (**C,D,G,H)**.

Binding of TN at high Ca^2+^ to the FITC-actin-WTTpm complex increased the value of Φ_E_ from 48.8 to 50.8° and θ_1/2_ from 6.2 to 7.2° (Fig. 3a,b). According to our earlier published data an increase in the Φ_E_ and θ_1/2_ values for FITC-actin is associated with global and/or local conformational changes that are accompanied by a turn of actin monomers away from the filament centre (Fig. 4A,C) and an increase in flexibility of F-actin in the thin filaments^4^. An increase in the flexibility of the thin filament correlates with F-actin shortening. TN at high Ca^2+^ induced an increase in the thin filament flexibility (reduced the persistence length of F-actin)^4^. A similar decrease in the persistence length of the thin filaments was observed at high Ca^2+^ by Isambert and coworkers^12^.

It was postulated that these changes in actin conformation were associated with a transition of the thin filament to the so-called “ON” state, and increased the fraction of actin monomers that facilitated the activation of the myosin ATPase^4,13,14^.

When ΔE139Tpm-TN complex was bound to F-actin, at high Ca^2+^ the values of Φ_E_ and θ_1/2_ increased from 48.7 to 51.7° and from 6.1 to 7.3°, respectively (Fig. 3a,b). This rise in the Φ_E_ and θ_1/2_ values was larger by 1.0 and 0.3°, respectively, with the mutant Tpm than with WTTpm (Fig. 4A,C). This may be interpreted as an increase in the fraction of the “switched on” actin monomers (ON state of the filament)^3,13^. Thus, the ΔE139 mutation increased the proportion of the “switched on” actin monomers in the thin filament at high Ca^2+^.

At low Ca^2+^ the values of Φ_E_ and θ_1/2_ for FITC-actin-WTTpm-TN were lower by 3.4° (47.4° vs 50.8°) and 1.5° (5.7° vs 7.2°), respectively, than the values of the correspondent parameters observed at high Ca^2+^ (Fig. 3). It is known that at low Ca^2+^ TnI is bound to F-actin^15,16^ and induces such conformational changes in F-actin, that lead apparently to a raised number of the “switched off” actin subunits^3,4^. It was postulated that in this case monomers were turned to the filament axis (rotated counterclockwise, Fig. 4B,D) as compared to their orientation in the “switched on” state^3,4^. The decrease in the value of θ_1/2_ is considered to be due to a reduction in the filament flexibility^4^. In turn, the latter was shown to be accompanied by an increase in the persistence length of the thin filament^12^.

Upon the binding of ΔE139Tpm-TN to F-actin at low Ca^2+^ the values of Φ_E_ and θ_1/2_ decreased from 48.7 to 46.8° and from 6.1 to 4.7°, respectively. Compared to WTTpm, the mutant Tpm showed a larger reduction in the Φ_E_ and θ_1/2_ values (Fig. 3a,b). This implies that at low Ca^2+^ both ΔE139Tpm and WTTpm caused a turn of actin monomers to the filament axis (counterclockwise rotation, Fig. 4B,D) and elongation of the thin filaments, with actin monomers being predominantly in the “switched off” state^3^. Thus, the ΔE139 did not interfere with the ability of TN to switch actin monomers between the “ON” and “OFF” states in thin filaments (Fig. 4).

### The E139 deletion shifts tropomyosin towards the inner domain of actin at low and high Ca^2+^

According to SDS–PAGE (Fig. 1B) the amount of the ΔE139Tpm bound was 85 ± 7% of the amount of Tpm found in fibres reconstructed with the WTTpm, likely reflecting the previously reported lower affinity of ΔE139Tpm for actin^6^. Incorporation of AF-WTTpm-TN or AF-ΔE139Tpm-TN into the actin filaments of the ghost fibres (Fig. 1) initiated polarized fluorescence^3,4^. The fluorescence intensity I_sum_ = (_⊥_I_⊥_ + _||_I_||_ + _||_I_⊥_ + _⊥_I_||_)/n, where n is the number of measurements, was 236 ± 15 and 266 ± 18 (n = 25) for the AF-WTTpm and AF-ΔE139Tpm, respectively. This implies that in the ghost fibres the fluorescence intensity of the mutant tropomyosin did not differ from that of the wild-type tropomyosin. Thus, the deletion had no significant effect on the number of tropomyosin molecules associated with the thin filaments in muscle fibres.

As illustrated in Fig. 3c,d, the binding of TN + Ca^2+^ to the F-actin-AF-WTTpm complex resulted in a decrease in the values of Φ_E_ from 55.7 to 53.4° and an increase in the values of θ_1/2_ from 3.5 to 4.5°. In our previous works^3,4^ and here, we relied on the observation done in the electron microscopy studies^5^ about the azimuthal shifting of Tpm strands relative to the outer and inner actin domains in different regulatory states of the thin filament. An increase in the Φ_E_ value for 5-IAF-labelled Tpm has been associated with such transition between the regulatory states when Tpm shifted to the outer domains of actin. On the contrary, a decrease in this value corresponded to the shifting of Tpm to the inner domains of actin subunits^3,4^.

An essential point is the correlation between the changes in the values of Φ_E_ and θ_1/2_ for 5-IAF-labelled Tpm (Fig. 3c,d). θ_1/2_ is an index of the flexibility of Tpm. An increase in flexibility was shown to correlate with shortening of Tpm, while a decrease in flexibility – with its elongation^17^. The decrease in the value of Φ_E_ was consistent with Tpm movement towards the thin filament axis (to the inner domains of actin subunits) as would be predicted by the shortening of the protein. It is to be noted that larger changes in θ_1/2_ correlated with larger alterations in Φ_E_ (Fig. 3c,d). Thus, the changes in the values of Φ_E_ and θ_1/2_ contain information about how far and in which direction the Tpm strands shift. The decrease in the value of Φ_E_ observed at binding of TN + Ca^2+^ to F-actin (Fig. 3c) could be explained by a relocation of Tpm towards the inner domain of actin to the closed position (Fig. 4C). Similar azimuthal shifts of the Tpm strands at binding of TN + Ca^2+^ to F-actin were observed earlier^5^.

At low Ca^2+^ the values of the angles Φ_E_ and θ_1/2_ for the F-actin-AF-WTTpm complex were 3.9° higher and 1.3° lower, respectively, as compared to the same parameters at high Ca^2+^. It seems plausible that the decrease in the value of θ_1/2_ for AF-WTTpm reflects an elongation of tropomyosin that would make it to move towards the outer domains of actin^5^.

We postulated that the Glu139 deletion moves the Tpm strands to the inner domain of actin (Fig. 4). Indeed when ΔE139Tpm was bound to F-actin, at transformation from the “ON” to “OFF” state the values of θ_1/2_ for FITC-actin and AF-ΔE139Tpm decreased by 36% and 17%, respectively, showing that the elongation of the mutant Tpm was smaller than that of F-actin. According to our suggestion, the mutant Tpm was expected to shift to the inner actin domain, as opposed to the WTTpm that moved towards the outer actin domain. The changes in the values of Φ_E_ for AF-ΔE139Tpm do not contradict this assumption. According to Fig. 3c, in the “OFF” state the value of Φ_E_ was lower for ΔE139Tpm than for WTTpm (by 1.7°), which agrees well with the putative movement of the mutant Tpm to the inner domain of actin in the thin filament (Fig. 4C,D). Thus, our assumption seems to be correct.

Our data indicated that the E139 deletion altered the ability of TN to shift Tpm in a Ca^2+^-dependent manner (Fig. 3c,d). Indeed, at low Ca^2+^ instead of shifting Tpm towards the outer domain of actin, to the blocked position (where Tpm would prevent myosin heads to bind strongly with F-actin^5^) TN moved the mutant tropomyosin towards the inner domain of actin, to the closed position (Fig. 4C). As in the closed position Tpm strands allow strong binding of the myosin heads to F-actin^5^, it is suggested that the shifting of the ΔE139Tpm to the closed position at low Ca^2+^ can contribute to the increase in the Ca^2+^-sensitivity observed earlier^7^ and here (Fig. 2). There is evidence that at low Ca^2+^, TnI bridges adjacent actin subunits across the filament^18^ and the residues 157–163 of TnI interact with the residue 146 of Tpm on the opposite actin helix^19^. This interaction induces Tpm movement toward the blocking position on actin^18^. Since residues 139 and 146 are close to one another, deletion of residue 139 might alter the TnI interaction with Tpm in a way that could restrain Tpm movement to locations near the closed position.

Thus, the E139 deletion in Tpm did not inhibit the ability of TN to switch actin monomers “on” and “off”, but made TN unable to move Tpm strands towards the blocked position at low Ca^2+^ (Fig. 4). Being located between the blocked and closed positions it could not block the binding of myosin to actin^5^. In addition, the mutation ΔE139 dramatically increased the flexibility of the thin filament: even at low Ca^2+^ concentrations, the flexibility of the mutant tropomyosin was more than 2 times higher than the flexibility of the WTTpm (Fig. 3d). With such a high degree of flexibility, ΔE139Tpm most likely cannot provide blocking of the myosin binding site on actin. Consequently, the abnormal position and high flexibility of the ΔE139Tpm (Fig. 3c) may be one of the reasons for a high Ca^2+^-sensitivity of the thin filament containing the mutant Tpm.

Our binding data (Fig. 1B) shows that incorporation of ΔE139Tpm into ghost fibres was 85 ± 7% of the amount of Tpm found in fibres reconstructed with WTTpm.Thus sections of actin filaments free of tropomyosin, may also contribute to the increase in Ca^2+^-sensitivity of the thin filaments.

It is clear that if the areas of the thin filament, where F-actin is not covered by Tpm are relatively large, it is difficult to expect not only significant differences between the WTTpm and ΔE139Tpm in the character of the changes in the parameters of polarized fluorescence, but also significant Ca^2+^-dependent changes in these parameters. In our experiments, significant differences between the WTTpm and ΔE139Tpm in the values for polarized fluorimetry parameters were found (Fig. 3). Thus in the ghost fibres, the thin filaments for a considerable length contain the tropomyosin-troponin complex and the differences between the ΔE139Tpm and WTTpm in the extent of their incorporation into the thin filaments are absent or insignificant.

Since we have data on the polarized fluorescence of FITC-actin in the ghost fibres before tropomyosin reconstitution (Fig. 3, Supplementary Table S1), it is easy to estimate a possible error introduced by the tropomyosin-free areas. Given that the extension of these areas is larger by ~15% in the thin filaments reconstituted with ΔE139Tpm, than for those reconstituted with WTTpm, this would slightly (by 0.3°) increase the Ф_E_ value for FITC-actin at low Ca^2+^. This means that the regions free of tropomyosin are able to destabilize the “OFF” state of the thin filaments. However, the changes in the parameter are small. Consequently, even if thin filaments reconstructed with ΔE139Tpm contained ~15% more regulatory units uncovered by Tpm than did filaments reconstituted with WTTpm, it would not distort the interpretation of the data.

Thus, the destabilization of the “OFF” state of the thin filaments containing ΔE139Tpm (Fig. 3) is largely related to the area of the filaments containing the troponin-tropomyosin complex. The Tpm-free regions of the thin filament can also contribute to an increase in the ATPase activity of actomyosin. Their contribution will depend on their extension. In our experiments the size of the regions containing the ΔE139Tpm was significantly larger than of those free of tropomyosin. However, both the regions of the thin filaments, containing the ΔE139Tpm (Fig. 3) and free of tropomyosin^6^ are capable of destabilizing the “OFF” state of the thin filaments.

### The E139 deletion alters the ability of the myosin heads to induce a shift of tropomyosin, switch actin monomers “on” and bind strongly to F-actin

The strong binding of the myosin heads to F-actin (AM state) occurs when S1 incorporates into the ghost fibres in the absence of nucleotides. At high Ca^2+^, in the presence of the WTTpm in the thin filament, strongly bound S1 caused an increase in the Φ_E_ value by 1.4° (Fig. 3a) and a decrease in the θ_1/2_ value by 0.7° (Fig. 3b) for FITC-actin, and a decrease in the Φ_E_ value by 2.6° (Fig. 3c) and an increase in the θ_1/2_ value by 0.8° (Fig. 3d) for AF-WTTpm. Judging by the character of these changes, F-actin upon its strong binding with S1 undergoes such conformational alterations that result in switching of actin monomers “on”^3,4^.

At high Ca^2+^, FITC-actin in the ghost fibres containing S1 showed by 0.4° higher values for each Φ_E_ and θ_1/2_ for the filaments containing ΔE139Tpm than for those containing WTTpm (Fig. 3a,b). This implies that the mutation increased the amount of the “switched on” actin monomers and shortened the thin filaments^4^. In parallel experiments, for AF-ΔE139Tpm the value of Φ_E_ decreased by 0.6° and the value of θ_1/2_ increased by 4.2°, showing that the ΔE139Tpm shifted towards the inner domain of actin and was shorter than WTTpm (Fig. 3c,d).

According to our data ΔE139Tpm moved further than WTTpm to the filament centre in response to S1 binding to the thin filaments (Fig. 4E,G). The larger shift of the mutant Tpm towards the filament centre contributed to a more pronounced increase in the number of the “switched on” actin monomers as compared to the case with WTTpm (Fig. 3a–d). Nevertheless, the relative amount of the myosin heads strongly bound to F-actin appeared to decrease in the presence of ΔE139Tpm, as judged by the fact that the value of Φ_E_ for AEDANS-S1 was by 0.4° higher for the fibres containing ΔE139Tpm then for those containing WTTpm (Fig. 3e). Such increase in the value of Ф_E_ can be interpreted as a decrease in amount of the myosin heads strongly bound to F-actin in the ghost muscle fibres^3,4^; it is possible to suggest that, despite tropomyosin’s location close to the open position, the mutant Tpm (unlike its wild-type counterpart^3^) was unable to facilitate the strong binding of the myosin heads to F-actin.

It was observed earlier that Tpm increases the amount of the myosin heads strongly bound to F-actin and shortens the Tpm strands in the thin filaments^3^; the deletion of the E139 residue in Tpm inhibited these effects (Fig. 3c,d). Similar changes were obtained earlier in the absence of troponin^20^. It was shown that in TN-free filaments, containing the ΔE139Tpm the latter was also shifted towards the inner domain of actin, with the amount of the myosin heads strongly bound to F-actin being lower than in the presence of WTTpm. Since it is known that myosin can bind to Tpm (with approximately 16 new links arising between the proteins, with the E139 residue being involved in one of them^20^), the perturbation in the Tpm molecule induced by E139 deletion might lead to changes in quantity and character of these links which would alter the binding of the myosin heads to Tpm^4^. Thus, the mutation in TPM2 induces such perturbation in Tpm molecule that alters its binding with S1, which inhibits the strong binding of the myosin heads to F-actin. Troponin has no effect on this interaction at high Ca^2+^.

Thus, the ΔE139 mutation induced a shift of the Tpm strands towards the inner domain of actin and did not inhibit the ability of TN to switch actin monomers “on” (Fig. 3a,c). A similar effect was observed earlier for the R167H and K168E mutations in TPM1^4^ and was not revealed for the R91G mutation in TPM2^20^ and A155T mutation in TPM1^19^. Thus, the E139 deletion altered tropomyosin’s ability to amplify the formation of the strong binding of S1 to F-actin either in the absence or in presence of TN and Ca^2+^.

### The effect of the nucleotides on behavior of the mutant tropomyosin and the response of actin and myosin

Binding of ADP, AMPPNP and ATP induced such conformational changes in F-actin that reduced the number of the “switched on” actin monomers (the values of Φ_E_ and θ_1/2_ for FITC-actin decreased, Fig. 3a,b), shifted WTTpm to the outer domains of actin (the values of Φ_E_ increased and the values of θ_1/2_ slightly decreased for AF-WTTpm, Fig. 3c,d) and decreased the amount of the myosin heads strongly bound to F-actin (the values of Φ_E_ and N increased for AEDANS-S1, Fig. 3e,f).

The E139 deletion significantly affected the position of the Tpm strands and the conformational state of the myosin heads and actin induced by the Tpm movement during the different stages of ATPase cycle at high Ca^2+^. In particular, with ΔE139Tpm, the Φ_E_ and θ_1/2_ values for FITC-actin (Fig. 3a,b), as well as the Φ_E_ and N values for AEDANS-S1 (Fig. 3e,f) were higher, whereas the Φ_E_ values were lower and θ_1/2_ values were higher for AF-ΔE139Tpm (Fig. 3c,d), at most stages of the ATPase cycle, as compared with the corresponding parameters obtained with WTTpm. Consequently, the amount of the “switched on” actin monomers was higher but the amount of the myosin heads strongly bound to F-actin was lower for the fibres containing ΔE139Tpm than for those reconstructed with WTTpm. Moreover, the ΔE139Tpm was shifted towards the inner domain of actin because the values of Φ_E_ for AF-ΔE139Tpm were lower than the corresponding values for the AF-WTTpm even in the presence of ATP (the values of Φ_E_ for AF-ΔE139Tpm were lower by 1.0° than for AF-WTTpm, Fig. 3c).

Thus, despite the fact that the mutant Tpm was located close to the inner domain of actin, the amount of the myosin heads strongly bound to F-actin for ΔE139Tpm was low (Figs 3e and 4E,G). This implied that the ΔE139 mutation inhibited the strong binding of the myosin heads to F-actin throughout the ATPase cycle at high Ca^2+^. A similar inhibition of the strong binding between the myosin heads and F-actin was observed earlier for the TN-free ghost fibres containing the ΔE139Tpm^20^. We suggested that the deletion of the E139 residue caused a perturbation in the Tpm molecule, which reduced the ability of tropomyosin to enhance the formation of strong binding of myosin heads to F-actin during the ATPase cycle (Fig. 4E,G). This inhibition is most likely due to the violation of assumed electrostatic binding between the E139 residue in Tpm and the K136 residue of the myosin head^21^. Since a decrease in the amount of the strongly bound myosin heads induces an inhibition of force production by myosin cross-bridges, it is possible that the perturbation in Tpm structure can induce the muscle weakness that was observed at myopathy associated with the ΔE139 deletion^6^.

The binding of S1 to F-actin-Tpm-TN at low Ca^2+^, on the contrary, induced a significant reduction in the values of Φ_E_ and θ_1/2_ for FITC-actin (Fig. 3a,b) and a decrease in Φ_E_ (Fig. 3c) and an increase in θ_1/2_ value (Fig. 3d) for AF-WTTpm at mimicking various intermediate stages of the ATPase cycle, showing that the amount of the “switched on” actin monomers decrease and WTTpm shifts towards the outer domain of actin^3,4^. As expected for the strong binding of the myosin heads, the values of Φ_E_ and N forAEDANS-S1 were higher than at high Ca^2+^ (Fig. 3e,f) indicating that the amount of the strongly bound myosin heads was lower^3,4^.

At low Ca^2+^ the ΔE139 mutation also essentially decreased the amount of the “switched on” actin monomers (the Φ_E_ and θ_1/2_ values decrease, Fig. 3a,b) but instead of a decrease in the amount of the myosin heads strongly bound to F-actin and a movement of the mutant Tpm towards the outer domain of actin, a shift of the ΔE139Tpm towards the open position took place (the Φ_E_ and θ_1/2_ values decreased, Fig. 3c,d) and the amount of the myosin heads strongly bound to F-actin increased (the Φ_E_ and N values decreased, Fig. 3e,f) during the ATPase cycle. It is necessary to note that a rise in the number of the myosin heads strongly bound to F-actin was observed even in the presence of ATP (Fig. 3e,f). Thus, at low Ca^2+^ the ΔE139Tpm was located closer to the inner domain of actin and the amount of the myosin heads strongly bound to F-actin were higher than for WTTpm, showing that a high Ca^2+^-sensitivity may result from the specific position taken by the mutant Tpm (Fig. 4F,H). An increase in the number of the myosin heads strongly bound to F-actin in the presence of ATP can inhibit the relaxation state leading to an appearance of the contracture and the muscle weakness.

## Conclusion

A major advantage of our approach involving the use of the ghost fibres over previous studies using isolated filaments from muscle fibres^6,7^ is that the thin filaments are arranged in much the same way as in an intact sarcomere and introduction of fluorescent probes allows to investigate the conformational changes in any of contractile and regulatory proteins at modeling muscle contraction^3,4^. The application of reconstituted muscle fibres has enabled us to reveal unknown details of regulation of actin-myosin interaction by troponin-tropomyosin complex during the ATPase cycle in the muscle fibres, containing the WTTpm or ΔE139Tpm. Our data have shown that Ca^2+^-regulation of actin-myosin interaction is mediated by conformational changes in troponin-tropomyosin complex and actin that result in spatial rearrangement and alterations in flexibility of these proteins. At high Ca^2+^, TN induces changes in conformation of Tpm that are accompanied by a slight increase in flexibility reflecting a decrease in the persistence length of the Tpm strands. At the same time, TN induces conformational changes in F-actin that turn the actin monomers towards the periphery of the thin filament and increase the flexibility of F-actin, indicating a pronounced decrease in the persistence length of F-actin (actin monomers are in the “switched on” state). At low Ca^2+^, TN conversely makes actin monomers turn to the outer of actin domain and slightly decreases the flexibility of F-actin, i.e. only slightly increases the persistence length of actin filament (actin monomers are “switched off”). The flexibility of Tpm decreases strongly, indicating a marked elongation of the Tpm strands.

We suggested that a change in position of the Tpm strands relative to the inner domain of actin may be associated with a disparity in the alterations in the persistence length of Tpm and F-actin that presumably cause azimuthal shifting of the Tpm strands. For example, if the Tpm strands at transition from the “ON” to the “OFF” state undergo a greater elongation than does F-actin, it may cause an azimuthal shift of the Tpm strands towards the outer domain of actin. Conversely, a lower (compared to F-actin) shortening of the Tpm strands move them to the inner domain of actin.

The conformational changes in troponin-tropomyosin complex and F-actin initiated by Ca^2+^ are interdependent^3^, therefore a point mutation in any of these proteins should disrupt this interdependency and induce deregulations of actin-myosin interaction. Our work demonstrates that the ΔE139 mutation induces such uncoupling. Indeed, TN keeps its ability to “switch off” actin monomers at low Ca^2+^ (Fig. 3a,b), but loses the ability to move Tpm strands towards the outer domain of actin (to increase the persistence length of the Tpm strands) (Fig. 3c,d), and this may contribute to the high Ca^2+^-sensitivity (Fig. 2). The regions of the thin filaments free of Tpm, can also activate ATPase, regardless of the concentration of Ca^2+^, and this causes destabilization of the “OFF” state^6^. However, these regions in our experiments are small.

The mutation also may alter the ability of Tpm to control the formation of the strong binding of the myosin heads to F-actin throughout the ATPase cycle; the amount of the myosin heads strongly bound to F-actin at mimicking of the AM stage decreases at high Ca^2+^, while increasing at low Ca^2+^ (Fig. 3e,f).

A high Ca^2+^-sensitivity was observed earlier for other mutations^6,7,19^. However, for the A155T substitution in TPM1 it was shown that the mutation inhibited also the TN’s ability to switch actin monomers “off” at low Ca^2+^ concentration^22^. The mutation R167H in TPM1^4^ retained (but slightly reduced) the ability of TN to move the Tpm strands towards the outer domain of actin but essentially depressed the ability of TN to switch actin monomers “off”. Thus, a mutation may increase the Ca^2+^-sensitivity in different ways. Therefore, it is helpful to know the molecular mechanism determining high Ca^2+^-sensitivity for each hypercontractile mutation.

## Methods

### Using of experimental animals

All experiments were performed on skinned muscle fibres and proteins from skeletal muscles of rabbit (Oryctolagus cuniculus). The animals were killed in accordance with the official regulations of the community council on the use of laboratory animals by the methods described earlier^4^. The study was approved by the Animal Ethics Committee of the Institute of Cytology of the Russian Academy of Science (Assurance Identification number F18-00380, valid until 31.10.2022).

### Preparation of proteins

Myosin and actin from fast skeletal rabbit muscles were prepared according to Margossian and Lowey^23^ and Spudich and Watt^24^, respectively. S1 was prepared by α-chymotrypsin digestion of rabbit myosin^25^. The reactive residue Cys707 of S1 was modified with 1,5-IAEDANS^26^. TN was isolated from fast rabbit skeletal muscle according to the method of Potter^27^. ββ-homodimers of recombinant wild-type tropomyosin Tpm2.2 (WTTpm) and with the E139 deletion (ΔE139Tpm) were expressed in BL21 (DE3) cells and purified as described before^28^. All Tpms had an N-terminal extension of two additional amino acids (AlaSer), which compensated for the reduced affinity of recombinant non-acetylated skeletal Tpm to F-actin^29^. Labelling of Tpm with 5-IAF at cysteine residues was performed as described previously^30^. The purity of the proteins was examined by SDS-PAGE.

### Determination of actin-activated ATPase of subfragment-1

The rate of the ATPase reaction was determined for fully regulated reconstituted thin filaments in a solution containing 1 μM S1, 7 μM F-actin, 3 μM troponin, 3 μM WTTmp or ΔE139Tpm in the following buffer: 12 mM Tris-HCl (pH 7.9), 2.5 mM MgCl_2_, 15 mM KCl, 20 mM NaCl, 0.2 mM dithiothreitol and 2 mM ATP at 25 °C. The reaction was carried out at Ca^2+^ concentrations increasing from 1 × 10^−9^ M to 1 × 10^−4^ M. The concentration of free Ca^2+^ in the presence of 2 mM EGTA was calculated using the Maxchelator program. The reaction was stopped after 10 min by adding trichloroacetic acid to a final concentration of 5%. The amount of inorganic phosphate formed was determined by the method of Fiske and Subarrow^31^. Three experiments were conducted. Statistical processing of data, calculation of the pCa_50_ value and plotting was carried out using GraphPad Prism 5.0 software.

### Preparation and labelling of ghost fibres

The bundles of about 100 fibres were separated from *m. psoas* of rabbit and placed into a cooled solution containing 100 mM KCl, 1 mM MgCl_2_, 67 mM K, Na phosphate buffer, pH 7.0, and 50% glycerol. Single fibres were gently isolated from the glycerinated musclebundle and incubated during 70–90 min in the solution containing 800 mM KCl, 1 mM MgCl_2_, 10 mM ATP, 6.7 mM K, Na phosphate buffer, pH 7.0^4^. Such fibres so called as ghost fibres consist of more than 80% of actin^3^. Thin filaments were reconstructed with Tpm (WTTpm or ΔE139Tpm) and TN and decorated with S1 by incubating of the fibre in a solution containing 50 mM KCl, 3 mM MgCl_2_, 1 mM DTT, 6.7 mM K, Na phosphate buffer, pH 7.0 and the corresponding proteins. The proteins that did not bind with F-actin were removed by the washing of the fibre in the same solution without proteins. FITC-phalloidin was dissolved in methanol and conjugated with F-actin of the fibres as described before^4,20^. The final composition of the fibres was examined using 12% SDS-PAGE gels, stained with Coomassie brilliant blue R (Sigma-Aldrich). The gels were scanned using the Kyocera scanner FS-1025 and the ratio of WTTpm to the mutant Tpm that bound to actin was determined by ImageJ 1.48 software. The molar ratios of S1 to actin were 1:5 (±2) in the absence of nucleotide and 1:5 (±2), 1:8 (±2), and 1:14 (±2) in the presence of ADP, AMPPNP, and ATP, respectively.

### Fluorescence polarization measurement

Steady-state polarized fluorescence was measured in ghost fibres using a flow-through chamber and polarized fluorimeter as described in details before^11^. The excitation wavelength was 436 ± 5 nm for 5-IAF associated with Tpms and 407 ± 5 nm for 1,5-IAEDANS bound with S1 and for FITC-phalloidin conjugated with F-actin. The intensity components (I) of polarized fluorescence were recorded at 500–600 nm with use of two photomultiplier tubes from 20–55 sites (5−11 fibres). Fluorescence polarization ratios were defined as: P_||_ = (_||_I_||_ − _||_I_⊥_)/(_||_I_||_ + _||_I_⊥_) and P_⊥_ = (_⊥_I_⊥_ − _⊥_I_||_)/(_⊥_I_⊥_ + _⊥_I_||_). The subscripts _||_ and _⊥_ designate the direction of polarization parallel and perpendicular to the fibre axis, the former denoting the direction of polarization of the incident light and the latter that of the emitted light.

Highly ordered arrangement of the fluorescent probes bound to the proteins in muscle fibre results in the appearance of polarized fluorescence and allows examining the average orientation of the probe and their mobility. According to the helix plus isotropic model^8–11^, the absorption and emission of light are realized by linear, completely anisotropic absorption (A) and emission (E) dipoles. The axes of dipoles of the ordered probes are spirally located along the surface of the cone, whose axis coincides with the long axis of the thin filament. In muscle fibre there are two populations of fluorophores–randomly distributed fluorophores (N) oriented at the magic angle of 54.7°, and the fluorophores located in a spiral (1–N) at an angle not equal to 54.7°. The former are not included in the population of the orderly oriented fluorophores (1–N), and are considered as randomly oriented (which contributes to N) indicating the mobility of the labelled protein. The changes in the angle of absorption (Φ_A_) and emission (Φ_E_) dipoles of oriented fluorophores contain qualitative information about a magnitude and direction of the spatial rearrangements of the labelled protein or its substantial part. The changes in microenvironment of the probes do not contribute to the probe movements because any reliable shifts of the spectrum of the proteins modified by 5-IAF, 1,5-IAEDANS or FITC-phalloidin were not detected. The position of the maximum of the fluorescence spectrum in all the experiments were measured with an accuracy of 0.3 nm^4,20^. The character of the Φ_A_ and Φ_E_ changes coincides with each other, so the only one of these values is presented in Results and Discussion. It is postulated that the thin filament is flexible and deviates from the fibre axis with the maximal angle θ_1/2_^10^. The values of sin^2^θ, Φ_A_ and Φ_E_ are fit by mathematical analysis to give the best agreement with the observed values of the intensities ratios _⊥_I_⊥_/_||_I_||_, _||_I_⊥_/_||_I_||_ and _⊥_I_||_/_||_I_||_^8–11^. Statistical significance of changes was evaluated using Student’s *t*-test, *p* < 0.05.

Intermediate stages of ATPase cycle were modelled in the absence or presence of 2.5 mM ADP (3 mM MgCl_2_), 16 mM AMPPNP (8 mM MgCl_2_) or 5 mM ATP (18 mM MgCl_2_). Solutions containing ATP comprised also 10 mM creatine phosphate and 140 unit/ml creatine kinase.

## Electronic supplementary material

Supplementary Figure A

Supplementary Figure B

Supplementary Figure C

Supplementary Figure D

Supplementary Table S1

Supplementary Table S2

Supplementary Table S3
