# Supplementary material for: Molecular mechanisms of dysfunction of muscle fibres associated with Glu139 deletion in TPM2 gene
Source: Sci Rep. 2017 Dec 1;7:16797. doi: 10.1038/s41598-017-17076-9 (PMC5711931; doi:10.1038/s41598-017-17076-9)
Supplement: Supplementary file 3 — Supplementary Figure C [file 41598_2017_17076_MOESM3_ESM.doc]

**Molecular mechanisms of dysfunction of muscle fibres associated with Glu139 deletion in *TPM2* gene**

**Yurii S. Borovikov^1^, Nikita A. Rysev^1^, Olga E. Karpicheva^1^, Vladimir V. Sirenko^1^, Stanislava V. Avrova^1^, Adam Piers^2^& Charles S. Redwood^2^**

**Supplementary Figure C.** The full-length SDS-PAGE gel showing (from the right to the left) recombinant human fast skeletal β-tropomyosin (WTTpm), ghost fibres reconstituted with WTTpm and troponin (TN), and ghost fibres. The bands used in the Figure 1A are framed.


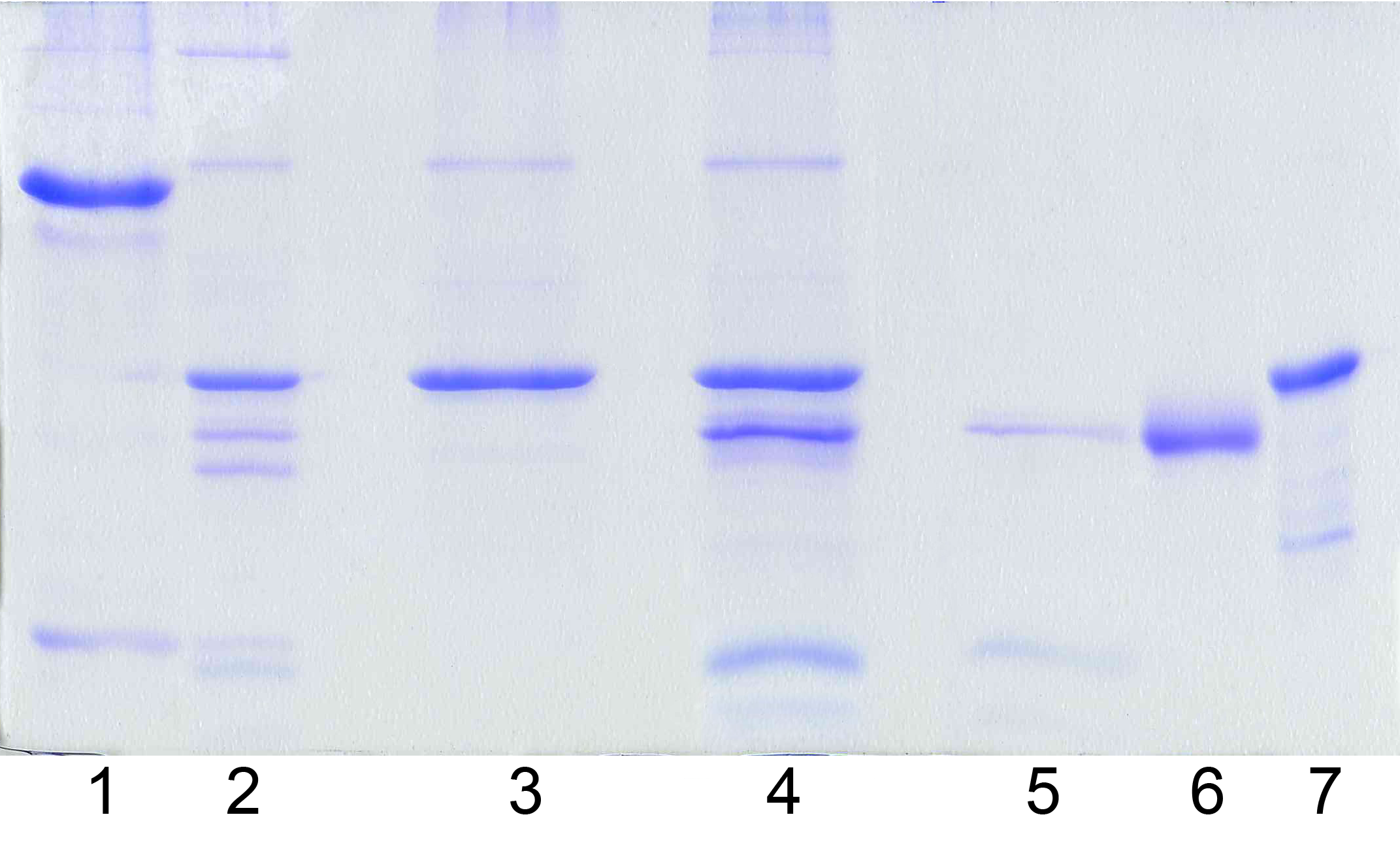


α-Actinin

Tpm2.2, TnT

LC1, TnI

TnC

Actin

Ghost Ghost fibres+ WTTpm

fibres +WTTpm+TN
