# Supplementary material for: Molecular mechanisms of dysfunction of muscle fibres associated with Glu139 deletion in TPM2 gene
Source: Sci Rep. 2017 Dec 1;7:16797. doi: 10.1038/s41598-017-17076-9 (PMC5711931; doi:10.1038/s41598-017-17076-9)
Supplement: Supplementary file 4 — Supplementary Figure D [file 41598_2017_17076_MOESM4_ESM.doc]

**Molecular mechanisms of dysfunction of muscle fibres associated with Glu139 deletion in *TPM2* gene**

**Yurii S. Borovikov^1^, Nikita A. Rysev^1^, Olga E. Karpicheva^1^, Vladimir V. Sirenko^1^, Stanislava V. Avrova^1^, Adam Piers^2^& Charles S. Redwood^2^**

**Supplementary Figure D.** The full-length SDS-PAGE gel showing rabbit fast skeletal actin (lane 1), muscle fibres (lane 2), WTTpm (lane 3), ghost fibres reconstituted with WTTpm (lane 4), ΔE139Tpm (lane 5), and ghost fibres reconstituted with ΔE139Tpm (line 6). The bands used in the Figure 1B are framed.


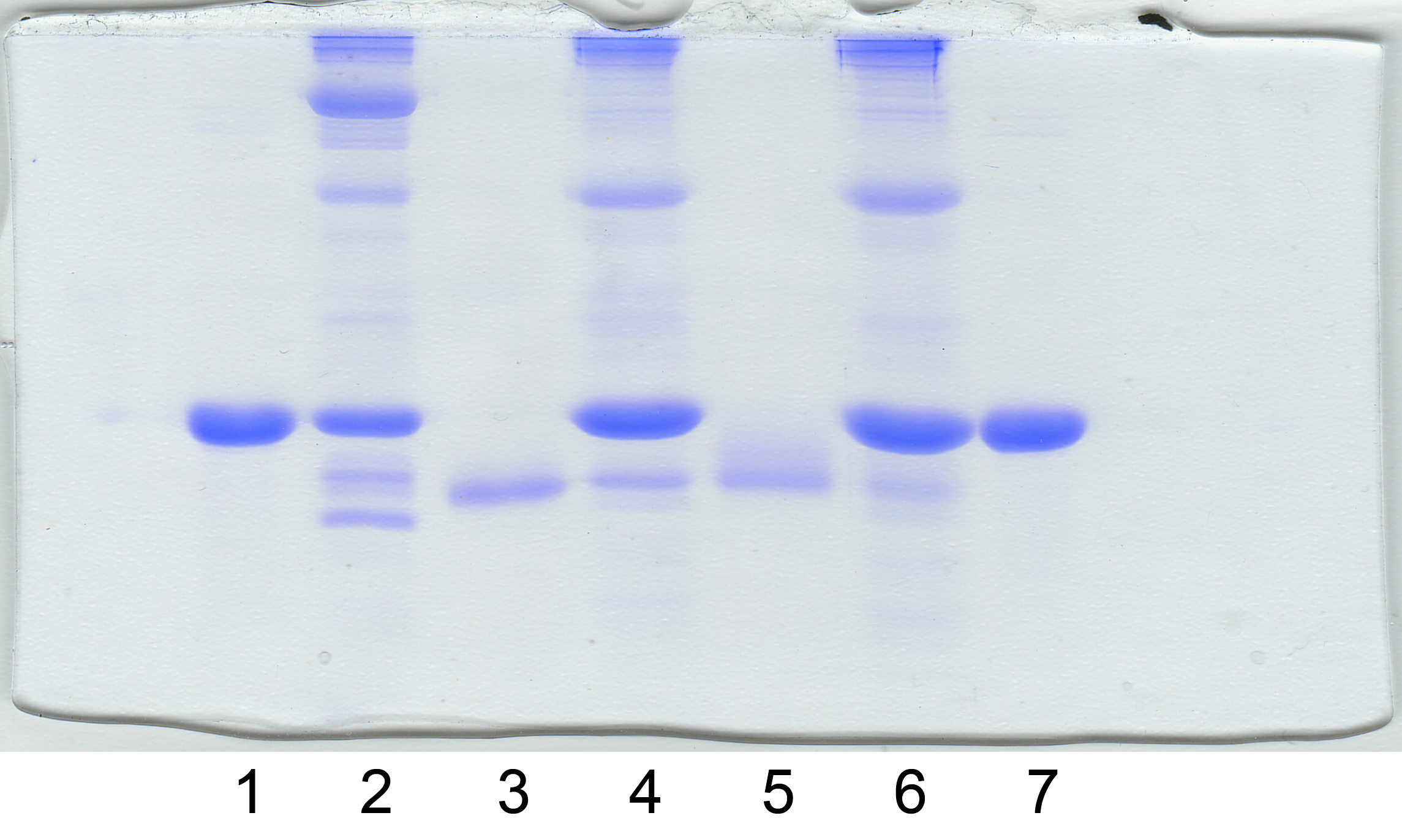


Tpm2.2, TnT

Tpm1.1

Actin

Myosin HC

MyBP-C

α-Actinin
