# Supplementary material for: Molecular mechanisms of dysfunction of muscle fibres associated with Glu139 deletion in TPM2 gene
Source: Sci Rep. 2017 Dec 1;7:16797. doi: 10.1038/s41598-017-17076-9 (PMC5711931; doi:10.1038/s41598-017-17076-9)
Supplement: Supplementary file 5 — Supplementary Table S1 [file 41598_2017_17076_MOESM5_ESM.doc]

**Molecular mechanisms of dysfunction of muscle fibres associated with Glu139 deletion in *TPM2* gene**

**Yurii S. Borovikov^1^, Nikita A. Rysev^1^, Olga E. Karpicheva^1^, Vladimir V. Sirenko^1^, Stanislava V. Avrova^1^, Adam Piers^2^ & Charles S. Redwood^2^**

**Supplementary Table 1.** The effect of TN (±Ca^2+^), S1, nucleotides and the wild-type (WT) and mutant (ΔE139) tropomyosins on polarization ratios of FITC-phalloidin bound to F-actin in ghost fibres.

| Nucleotide | TN | Ca^2+^ | S1 | WT | ΔE139 | P_║_ ± SEM | P_⊥_ ± SEM |
| --- | --- | --- | --- | --- | --- | --- | --- |
| - | - | - | - | - | - | 0.314± 0.001 | 0.094± 0.002 |
| - | - | - | - | + | - | 0.309± 0.002 | 0.125± 0.003 |
| - | - | - | - | - | + | 0.317± 0.001 | 0.115± 0.002 |
| - | + | + | - | + | - | 0.294± 0.002 | 0.185± 0.002 |
| - | + | - | - | + | - | 0.333± 0.002* | 0.109± 0.001 |
| - | + | + | - | - | + | 0.281± 0.002 | 0.210± 0.002 |
| - | + | - | - | - | + | 0.337± 0.002* | 0.048± 0.001 |
| - | + | + | + | + | - | 0.257± 0.002 | 0.207± 0.002 |
| - | + | - | + | + | - | 0.346± 0.002 | 0.055± 0.001 |
| - | + | + | + | - | + | 0.247± 0.003 | 0.227± 0.002 |
| - | + | - | + | - | + | 0.332± 0.002 | 0.048± 0.001 |
| ADP | + | + | + | + | - | 0.294± 0.002 | 0.138± 0.002 |
|  | + | - | + | + | - | 0.335± 0.001 | 0.067± 0.001 |
|  | + | + | + | - | + | 0.281± 0.002 | 0.143± 0.001 |
|  | + | - | + | - | + | 0.340± 0.002 | 0.014± 0.002 |
| AMP-PNP | + | + | + | + | - | 0.313± 0.001 | 0.109± 0.001 |
|  | + | - | + | + | - | 0.344± 0.002 | 0.064± 0.002 |
|  | + | + | + | - | + | 0.308± 0.003 | 0.074± 0.001 |
|  | + | - | + | - | + | 0.317± 0.002 | 0.042± 0.002 |
| ATP | + | + | + | + | - | 0.307± 0.003 | 0.110± 0.003 |
|  | + | - | + | + | - | 0.352± 0.002 | 0.018± 0.002 |
|  | + | + | + | - | + | 0.316± 0.003 | 0.093± 0.001 |
|  | + | - | + | - | + | 0.330± 0.002 | 0.035± 0.001 |

P_║_ and P_⊥_ measurements were performed as described in Materials and methods. The signs “+” and “–“ mean that the values were obtained in the presence and absence of TN, Ca^2+^, S1, nucleotides, and tropomyosins. The number of fibres used in each experiment was 6-8. TN (±Ca^2+^), S1, WT and mutant (ΔE139) tropomyosins and the nucleotides had pronounced effect on the values of P_||_ and P_^_, indicating the changes in the conformational state of F-actin in ghost fibers (p < 0.05). Asterisks indicate statistically insignificant difference in the values of P_║_ between the WT and ΔE139 tropomyosins.
