# Supplementary material for: Molecular mechanisms of dysfunction of muscle fibres associated with Glu139 deletion in TPM2 gene
Source: Sci Rep. 2017 Dec 1;7:16797. doi: 10.1038/s41598-017-17076-9 (PMC5711931; doi:10.1038/s41598-017-17076-9)
Supplement: Supplementary file 6 — Supplementary Table S2 [file 41598_2017_17076_MOESM6_ESM.doc]

**Molecular mechanisms of dysfunction of muscle fibres associated with Glu139 deletion in *TPM2* gene**

**Yurii S. Borovikov^1^, Nikita A. Rysev^1^, Olga E. Karpicheva^1^, Vladimir V. Sirenko^1^, Stanislava V. Avrova^1^, Adam Piers^2^ & Charles S. Redwood^2^**

**Supplementary Table 2**. The effect of TN (±Ca^2+^), S1 and nucleotides on polarization ratios of 5-IAF bound to the wild-type (WT) or mutant (ΔE139) tropomyosins in ghost fibres.

| Nucleotide | TN | Ca^2+^ | S1 | WT | ΔE139 | P_║_ ± SEM | P_⊥_ ± SEM |
| --- | --- | --- | --- | --- | --- | --- | --- |
| - | - | - | - | + | - | 0.046± 0.002 | 0.174± 0.003 |
| - | - | - | - | - | + | 0.137± 0.001 | 0.151± 0.002 |
| - | + | + | - | + | - | 0.074± 0.001 | 0.175± 0.002* |
| - | + | - | - | + | - | 0.039± 0.002 | 0.178± 0.001 |
| - | + | + | - | - | + | 0.179± 0.002 | 0.173± 0.002* |
| - | + | - | - | - | + | 0.167± 0.002 | 0.155± 0.001 |
| - | + | + | + | + | - | 0.110± 0.002 | 0.141± 0.002 |
| - | + | - | + | + | - | 0.061± 0.002 | 0.103± 0.001 |
| - | + | + | + | - | + | 0.184± 0.003 | 0.178± 0.002 |
| - | + | - | + | - | + | 0.167± 0.002 | 0.157± 0.001 |
| ADP | + | + | + | + | - | 0.105± 0.002 | 0.138± 0.002 |
|  | + | - | + | + | - | 0.055± 0.001 | 0.107± 0.001 |
|  | + | + | + | - | + | 0.190± 0.002 | 0.186± 0.001 |
|  | + | - | + | - | + | 0.169± 0.002 | 0.156± 0.002 |
| AMP-PNP | + | + | + | + | - | 0.021± 0.001 | 0.121± 0.001 |
|  | + | - | + | + | - | 0.011± 0.002 | 0.133± 0.002* |
|  | + | + | + | - | + | 0.141± 0.003 | 0.145± 0.001 |
|  | + | - | + | - | + | 0.146± 0.002 | 0.135± 0.002* |
| ATP | + | + | + | + | - | 0.081± 0.003 | 0.163± 0.003 |
|  | + | - | + | + | - | 0.008± 0.002 | 0.153± 0.002* |
|  | + | + | + | - | + | 0.196± 0.003 | 0.176± 0.001 |
|  | + | - | + | - | + | 0.180± 0.002 | 0.155± 0.001* |

TN (±Ca^2+^), S1, WT and mutant (ΔE139) tropomyosins and the nucleotides had pronounced effect on the values of P_||_ and P_^_, indicating the changes in the conformational state of tropomyosins in ghost fibres (p < 0.05). Designations are as in the legend to Supplementary Table 1. Asterisks indicate statistically insignificant differences in the values of P_^_ between the WT and ΔE139 tropomyosins.
