# Supplementary material for: Molecular mechanisms of dysfunction of muscle fibres associated with Glu139 deletion in TPM2 gene
Source: Sci Rep. 2017 Dec 1;7:16797. doi: 10.1038/s41598-017-17076-9 (PMC5711931; doi:10.1038/s41598-017-17076-9)
Supplement: Supplementary file 7 — Supplementary Table S3 [file 41598_2017_17076_MOESM7_ESM.doc]

**Molecular mechanisms of dysfunction of muscle fibres associated with Glu139 deletion in *TPM2* gene**

**Yurii S. Borovikov^1^, Nikita A. Rysev^1^, Olga E. Karpicheva^1^, Vladimir V. Sirenko^1^, Stanislava V. Avrova^1^, Adam Piers^2^ & Charles S. Redwood^2^**

**Supplementary Table 3**. The effect of TN (±Ca^2+^), nucleotides, wild-type (WT) or mutant (ΔE139) tropomyosins on polarization ratios of 1,5-IAEDANS bound to S1 in ghost fibres.

| Nucleotide | TN | Ca^2+^ | WT | ΔE139 | P_║_ ± SEM | P_⊥_ ± SEM |
| --- | --- | --- | --- | --- | --- | --- |
| - | - | - | + | - | 0.396± 0.001 | -0.048± 0.002* |
| - | - | - | - | + | 0.400± 0.001 | -0.050± 0.002* |
| - | + | + | + | - | 0.397± 0.002* | -0.049± 0.002 |
| - | + | - | + | - | 0.387± 0.003 | 0.003± 0.001 |
| - | + | + | - | + | 0.399± 0.002* | -0.011± 0.002 |
| - | + | - | - | + | 0.380± 0.003 | 0.025± 0.001 |
| ADP | + | + | + | - | 0.419± 0.002 | -0.029± 0.002 |
|  | + | - | + | - | 0.397± 0.001* | 0.043± 0.001* |
|  | + | + | - | + | 0.406± 0.002 | 0.003± 0.003 |
|  | + | - | - | + | 0.397± 0.002* | 0.043± 0.003* |
| AMP-PNP | + | + | + | - | 0.390± 0.002 | -0.049± 0.002 |
|  | + | - | + | - | 0.335± 0.002 | 0.104± 0.002 |
|  | + | + | - | + | 0.378± 0.002 | -0.012± 0.002 |
|  | + | - | - | + | 0.358± 0.002 | 0.046± 0.002 |
| ATP | + | + | + | - | 0.362± 0.002 | 0.151± 0.002 |
|  | + | - | + | - | 0.322 ± 0.002 | 0.234± 0.002 |
|  | + | + | - | + | 0.334± 0.002 | 0.204± 0.002 |
|  | + | - | - | + | 0.340± 0.001 | 0.176± 0.002 |

TN (±Ca^2+^), WT and mutant (ΔE139) tropomyosins and the nucleotides had pronounced effect on the values of P_||_ and P_^_, indicating the changes in the conformational state of S1 in ghost fibres (p < 0.05). Designations are as in the legend to Supplementary Table 1. Asterisks indicate statistically insignificant differences in the values of P_║_ and P_^_ between the WT and ΔE139 tropomyosins.
